# Constraint Based Modeling Going Multicellular

**DOI:** 10.3389/fmolb.2016.00003

**Published:** 2016-02-10

**Authors:** Patricia do Rosario Martins Conde, Thomas Sauter, Thomas Pfau

**Affiliations:** ^1^Systems Biology Group, Life Sciences Research Unit, Faculty of Sciences, Technology and Communications, University of LuxembourgLuxembourg, Luxembourg; ^2^Department of Physics, Institute of Complex Systems and Mathematical Biology, University of AberdeenAberdeen, UK

**Keywords:** multi-scale modeling, constraint based modeling, multi-tissue modeling, multi-organism modeling, metabolic modeling

## Abstract

Constraint based modeling has seen applications in many microorganisms. For example, there are now established methods to determine potential genetic modifications and external interventions to increase the efficiency of microbial strains in chemical production pipelines. In addition, multiple models of multicellular organisms have been created including plants and humans. While initially the focus here was on modeling individual cell types of the multicellular organism, this focus recently started to switch. Models of microbial communities, as well as multi-tissue models of higher organisms have been constructed. These models thereby can include different parts of a plant, like root, stem, or different tissue types in the same organ. Such models can elucidate details of the interplay between symbiotic organisms, as well as the concerted efforts of multiple tissues and can be applied to analyse the effects of drugs or mutations on a more systemic level. In this review we give an overview of the recent development of multi-tissue models using constraint based techniques and the methods employed when investigating these models. We further highlight advances in combining constraint based models with dynamic and regulatory information and give an overview of these types of hybrid or multi-level approaches.

## 1. Introduction

While genome sequences are now readily available, determining the metabolic properties of organisms is still an open problem. Numerous methods for modeling and analyzing metabolism exist (for a general comparison please refer to Bordbar et al., 2014), including a large diversity of multi-scale approaches. E.g., Karr et al. (2012) reconstructed a whole-cell model of *Mycoplasma genitalium*. The overall model combines multiple different modeling techniques for describing different levels of the organism, reaching from regulatory signaling to metabolism and other pathways. However, its comprehensiveness leads to a large number of parameters, making this approach practically quite challenging for more complex organisms. There also exist some applications of kinetic modeling to study different tissues, like liver (Ricken et al., 2015; Zeigerer et al., 2015), heart (Crampin et al., 2004), and brain metabolism (Jolivet et al., 2015), mainly in the framework of the Virtual Physiological Human Project (Viceconti et al., 2008). A hepatocyte model integrating signaling and regulatory information along with metabolism based on kinetic modeling has recently been published (Ryll et al., 2014). Petri-nets form another approach used for multi-scale modeling (Berestovsky et al., 2013). However, due to the availability of genome annotations and the lack of knowledge on kinetic parameters, stoichiometric network models of metabolism form an widely used and very scalable basis. The concept of constraint based modeling (CBM) allows fast calculations of large networks under the steady state assumption, relying mainly on genetic information easily obtainable by genome sequencing. Recent combinations of these types of models with other modeling techniques show promising results. We will therefore focus in this review on constraint based modeling and its combination with other techniques.

Constraint based modeling (CBM) aims at helping researchers to get a better insight into the complex system of metabolism (Llaneras and Picó, 2008). Additionally, they exploit the property of metabolism that reactions have defined substrates and products. Based on a network of biochemical reactions, a stoichiometric matrix *S* is created, with columns representing reactions, rows representing metabolites, and entries *S*(*i, j*) representing the stoichiometric coefficient of metabolite *i* in reaction *j*. For larger networks this requires the application of computational tools usually providing the models in SBML format being an important step toward reproducible science (Pfau et al., 2015). The major constraint imposed by CBM methods is the assumption of an internal quasi steady state of the investigated system. It is assumed that the internal concentrations of metabolites do not change over time (i.e., *S* · *v* = 0, where *v* is the flux distribution vector of the system). In addition, three primary constraints are introduced to obtain biologically relevant solutions (Orth and Palsson, 2010; Lewis et al., 2012): mass and charge conservation within reactions, dependency on substrate and enzyme availability and reversibility constraints based on thermodynamics. It is also common to employ an objective function that the system is assumed to be optimized for (Schuetz et al., 2007). The implemented functions range from growth (Feist and Palsson, 2010) over production of ATP to complex combinations of multiple simpler objectives (Vo et al., 2004) and are a particular challenge when multicellular organisms are the target of research. One of the most common approaches to CBM is the use of flux balance analysis (FBA) (Savinell and Palsson, 1992; Varma and Palsson, 1994; Kauffman et al., 2003; Raman and Chandra, 2009; Orth and Palsson, 2010). FBA has been used to investigate the effects of knockouts on metabolism (Segrè et al., 2002; Shlomi et al., 2005) and to design knockout strategies for metabolic engineering (Burgard et al., 2003; Rocha et al., 2010; von Kamp and Klamt, 2014). Basic FBA however, does not account for model dynamics like gene regulation, signaling processes, or metabolic regulation (Orth and Palsson, 2010). Therefore, approaches considering these regulatory elements have been developed (Covert and Palsson, 2002; Mahadevan et al., 2002; Covert et al., 2008). Models for multiple species from all biological kingdoms (prokaryotes, eukaryotes, and archaea) have been reconstructed (see http://systemsbiology.ucsd.edu/InSilicoOrganisms/OtherOrganisms), and their number is constantly increasing. While the first models used in CBM were mostly aimed at central carbon metabolism, the ever growing availability of genome sequences has let to a rapid increase in genome scale metabolic reconstructions (GSM). The first GSM was *Haemophilus influenzae Rd* (Edwards and Palsson, 1999) in 1999, followed four years later by the first eukaryotic model (*Saccharomyces cervisiae*) (Förster et al., 2003) and the first mammalian model (*Mus musculus*) in 2005 (Sheikh et al., 2005). The first human reconstruction (Recon1 by Duarte et al., 2007) was closely followed by the publication of a second human GSM, the Edinburgh Human Metabolic Network, within the same year (Ma et al., 2007). These initial models have seen many improved versions over the past years (e.g., Recon2 Thiele et al., 2013 and HMR2 Mardinoglu et al., 2014 for human). While general reconstructions serve as an important knowledge base for our understanding of metabolic capabilities within the reconstructed organism, they are limited when investigating multicellular organisms exhibiting multiple different tissues. Since different cell types of higher organisms have different functions, and indeed different capabilities, it is therefore necessary to reconstruct tissue specific models. In a recent review, Ryu et al. (2015) give an extensive overview over currently available reconstructions. In addition to manual reconstruction, multiple methods exist for contextualization of metabolic networks (reviewed in Machado and Herrgård, 2014; Resendis-Antonio et al., 2014; Robaina Estévez and Nikoloski, 2014; Ryu et al., 2015). While tissue specificity can help to elucidate important information about a specific tissue, a single tissue model on its own is unable to inform about the complex interactions occurring in a higher organism. This necessitates the combination of models presenting multiple tissues. In this review we will first provide an overview of methods developed for the integration of non-metabolic information in constraint based models and give an overview of methods used for model simulation. We will continue by detailing the recent development of constraint based models spanning multiple organisms or tissues along with methods employed when investigating these models. Finally we will present recent advances in combining constraint based models with dynamic and regulatory information and give an overview of these types of hybrid or multi-scale approaches.

## 2. FBA method: data integration and extensions

**Figure 1:**
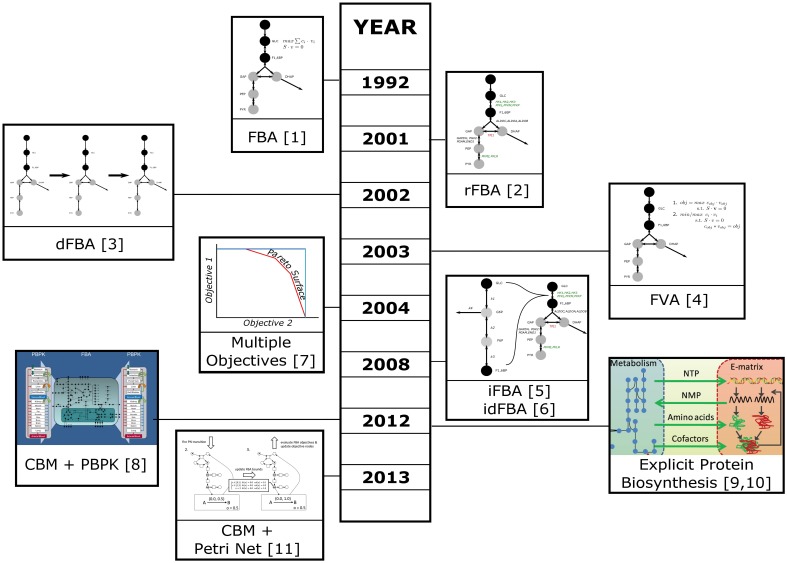
**Timeline of the development of techniques for the integration of data and the simulation and analysis of complex systems**. Please refer to the main text for details. ([1] Savinell and Palsson (1992); [2] Covert et al. (2001); [3] Mahadevan et al. (2002); [4] Mahadevan and Schilling (2003); [5] Covert et al. (2008); [6] Lee et al. (2008); [7] Vo et al. (2004); [8] Krauss et al. (2012); [9] Thiele et al. (2012); [10] Lerman et al. (2012); [11] Fisher et al. (2013)), Images for [8],[9], and [10] are derived from images taken from the respective publications which are provided under a Creative Commons attribution license (https://creativecommons.org/licenses/by/2.0/).

Multiple methods can be used to improve constraint based model predictions. Figure 1 provides an overview of these methods, ordered by their time of publication. These methods include ways to integrate regulatory events which can influence the activity of reactions by altering the production of specific proteins necessary to catalyze these reactions. They also provide concepts which allow the use of omics data to determine the availability of enzymatic activities. The first attempts to integrate non-metabolic information into a CBM analysis was introduced by Covert et al. (2001), who linked regulatory data to a CBM model of *Escherichia coli* turning reactions on and off (a method termed rFBA). The principle of flux adjustment based on external data was picked up by others and either used to switch reactions on or off (Becker and Palsson, 2008; Vlassis et al., 2014) or to adjust the bonds of fluxes (Colijn et al., 2009; Lee et al., 2012). The former try to derive the activity state of genes and assign reaction availabilities based on these activities using Boolean gene-protein-reaction association rules. These rules are similar to the Boolean rules used by Covert et al. for regulatory constraints, but only use gene activity information, where Covert et al. used additional information, like the availability of a preferred carbon source deactivating a second importer. These methods used for omics integration have received much attention lately and were extensively reviewed in Machado and Herrgård (2014) and Robaina Estévez and Nikoloski (2014). The reviews focused on flux bound adjustment and network structure contextualization, respectively.

Simultaneous to the development of rFBA, Mahadevan et al. introduced the concept of dynamic FBA (dFBA), which allows the simulation of time courses using FBA (Mahadevan et al., 2002). The idea is to use the resulting outputs (e.g., remaining amount of substrate, generated products) of an earlier time step as inputs for the next time step. Both concepts where later combined to form integrated dynamic FBA (iFBA or idFBA) which uses both time dependent regulatory information and the dFBA approach (Covert et al., 2008; Lee et al., 2008). In addition, Covert et al. used a small dynamic ODE model to dynamically simulate parts of the network based on the results of the earlier time step. This was also the first attempt to combine constraint based and dynamic models in a common framework. In iFBA the ODE model was solved to calculate initial rates, which were subsequently applied to the constraint based model as flux bounds. In addition, gene expression data for each time step was applied to the CBM restricting the active parts. Finally, the CBM was optimized and the resulting metabolite concentrations used as inputs to the next iteration of the ODE model.

Integration of different data types on the same model, requires the development of new optimization approaches. Each of these approaches is specifically designed for the research problem of interest. In Table 1, an overview on the main FBA-based methods discussed in this review is given. For a broader view on FBA methods, please refer to Lewis et al. (2012). Since 2012, other optimization methods have been developed, and we will briefly present some of them here. Pozo et al. (2012) developed a new optimization algorithm which allows to determine the global optimum of kinetic metabolic models while optimizing for multiple-objectives. Additionally, this algorithm allows the selection of sets of the most efficient optimal alternatives. This optimization approach can be very interesting when being applied to metabolic engineering, i.e., it can be applied when the objective is to optimize the synthesis rate of metabolite X, at minimum cost, and with minimum concentration change of intermediate metabolites. Recently, another approach, developed by Andreozzi et al. (2015), employs CBMs at steady-state and metabolite concentrations to derive feasible kinetic models which are representative of a specific physiological state. As the previous algorithm, this framework is easily applicable to metabolic engineering and can give hints about which enzymes to alter in order to achieve a specific physiological state. Furthermore, it can be used to decrease the uncertainty related with kinetic parameter estimation and to efficiently sample the solution space.

**Table 1:** **Overview of different FBA-based methods**.

**Approach**	**Concept**	**Description**	**Mathematical concepts**
FBA [1]	Optimize an objective function	Keep the internal system in steady state, and optimize a given objective using Linear Programming (LP)	*Min*/*Max w* = ∑ *c*_*i*_*v*_*i*_ s.t. *S* · *v* = 0 *lb*_*i*_ ≤ *v*_*i*_ ≤ *ub*_*i*_
rFBA [2]	Optimization of a metabolic and regulatory network	Transcription regulation integrated as time dependent constraints (0,1) using boolean rules to compare multiple time points.	FBA s.t. *v*_*i*_ = 0 if rule for reaction i inactive
dFBA-DOA [3]	Optimize multiple time-steps simultaneously	Simultaneous optimization over the entire time period	Non Linear Problem, combining end point objective and intermediate objectives
dFBA-SOA [3]	Optimize multiple time-steps iteratively	Divide the time period in intervals Use initial conditions or result from the previous time step to solve the dynamic equations Fix the metabolic model fluxes to the fluxes obtained when solving the ODEs Optimize the model at the next time step	FBA s.t. $lb_{i,t}=f_{i,lb}\left({\vec{v}}_{t-1}\right)$ $ub_{i,t}=f_{i,ub}\left({\vec{v}}_{t-1}\right)$ f includes changes to the initial conditions (i.e., available concentrations).
Thermodynamic FBA [4]	Integration of thermodynamical constraints into a metabolic network	Changing of reaction reversibility dependent on free energy changes	Adjust *ub*_*i*_ and *lb*_*i*_ according to thermodynamic considerations
iFBA [5]	Optimization of an integrated metabolic, regulatory and kinetic network	Combination of dFBA-SOA, and rFBA	
idFBA [6]	Optimization of an integrated metabolic, regulatory and signaling network.	dFBA-SOA with additional phenotypic data After each time step, update phenotype variables Constrain next time step using updated phenotype data	dFBA with additional time dependent constraints *v*_*t*, *i*_ = 0∀*i with I*_*t, i*_ = 0 I is the incidence matrix with one column per time point
ME-Matrix [7,8]	Optimization of a coupled metabolic and cellular machinery model	Addition of explicit biosynthesis of cellular machinery necessary for metabolic reactions Coupling constraints that couple protein synthesis to flux rate	Coupling: *v*_*i*_ − *c*_*max*_ · *v*_*s*_ ≤ 0 *v*_*i*_ − *c*_*min*_ · *v*_*s*_ ≥ −*s, s* ≥ 0 with *c*_*min, max*_ the coupling coefficient. and the first equation enforcing more protein synthesis for higher flux
QQSPN (Quasy-steady state Petri nets) [9]	Optimization of an integrated metabolic, regulatory and signaling network using Petri-nets	Calculate constraint and objective nodes for each time step Update the metabolic model according to the previous step Optimize the metabolic model Update the Petri net objective nodes according to the new objective	

*Integration of different approaches with CBMs lead to the development of new optimization frameworks. In this table, some FBA-based approaches are described*.

*c, optimization weight; Max, maximization; Min, minimization; lb, lower bound; s, accumulation variable; ub, upper bound; w, objective function*.

References: [1] Savinell and Palsson (1992); [2] Covert et al. (2001); [3] Mahadevan et al. (2002);[4] Beard et al. (2004); [5] Covert et al. (2008); [6] Lee et al. (2008); [7] Thiele et al. (2012); [8] Lerman et al. (2012); [9] Fisher et al. (2013)

Constraint based metabolic models are commonly aimed to simulate the metabolism of small molecules and tend to include macromolecular biosynthesis and modifications only implicitly by the inclusion of energy or reductant consuming reactions. Recently this neglect has been addressed in studies on *E.coli* and *Thermotoga maritima* which explicitly modeled the macromolecular biosynthesis machinery (Lerman et al., 2012; Thiele et al., 2012). The basis for this approach is a CBM model of the target organism, which was noted as M-matrix and a stoichiometric model of the macromolecular synthesis machinery, noted as E-matrix. The models are combined by forming a large ME matrix and adding coupling constraints that restrict the flux through reactions by requiring the catalyzing enzymes to be available. In addition, biomass is adjusted to reflect the explicit costs of macromolecular synthesis and amino acids, since proteins are allowed to accumulate in the model. In Thiele et al. (2012), the ME-model was simulated and the predictions matched with experimental growth rates and knockout phenotypes. Lerman et al. (2012) used the technique to investigate minimal ribosomal production rates necessary at specific growth rates, and could show changes consistent with experimental data. They also found pathways which become necessary for efficient growth in the ME-model but which are not important in a pure metabolic model. Thiele et al. (2012) mention that performing FBA in the ME-matrix is time consuming, therefore this approach does not easily scale to larger models. In practice, this dimensionality is likely to become too high for many eukaryotic models.

To tackle more complex systems the classic FBA formulation optimizing for a single objective is often insufficient. One approach to handle those multi-objective systems is to use a concept called pareto optimality, where a solution is searched for which any change would lead to the worsening of at least one objective (Oh et al., 2009). The concept has been applied to optimize a human mitochondrial model taking into consideration three objective functions (Vo et al., 2004). There are different ways to combine objectives, e.g., by weighted sums of objective functions, or by successive optimization and fixation of the objective value of one target flux. However, even when multiple objectives are employed, the final solution is not necessarily unique and the number of alternate optima might become so high that is infeasible to calculate them. To analyze those solutions it is often useful to perform Flux Variability Analysis (FVA; Mahadevan and Schilling, 2003). In contrast to FBA, FVA determines the allowed ranges of fluxes under the optimal conditions. This can help to indicate flexible reactions, with large ranges of possible flux values, and reactions under tight control, which show only a very small range of possible fluxes.

## 3. Combining multiple constraint based models

**Figure 2:**
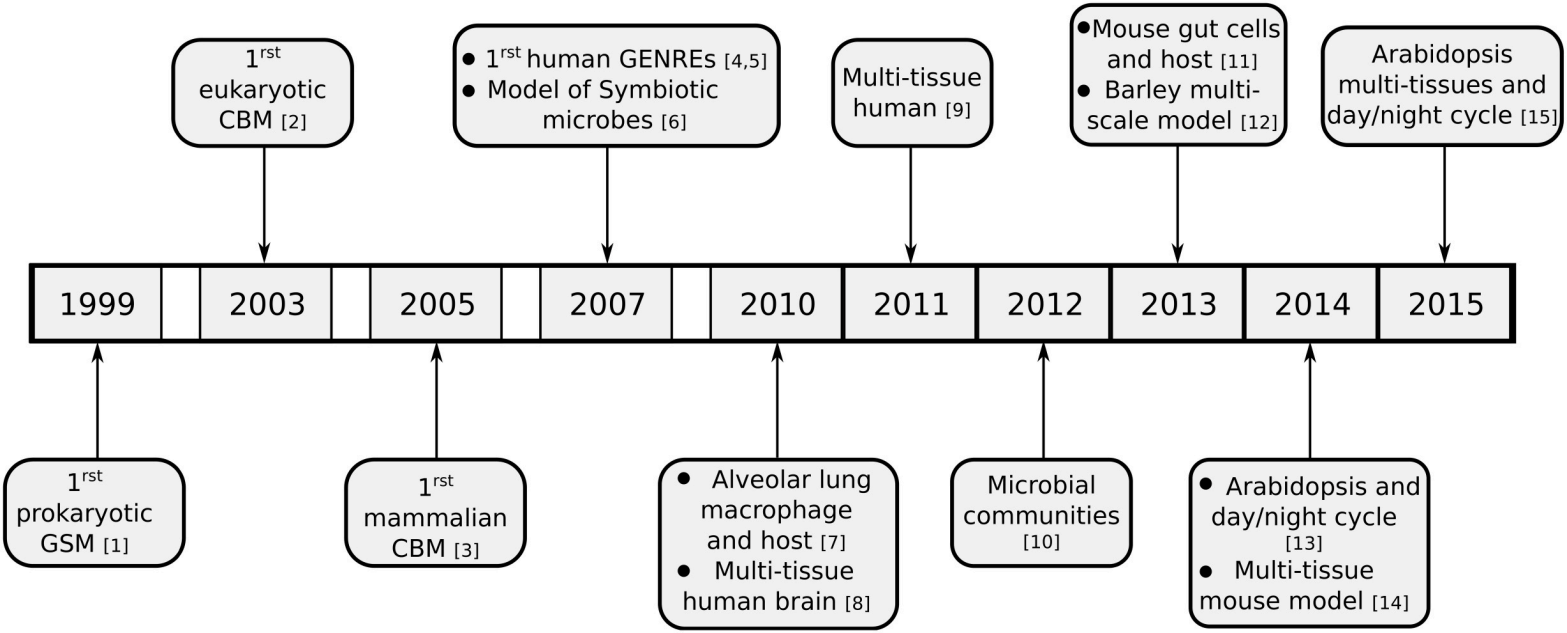
**Timeline of development of reconstruction of metabolic models and realization of different models spanning multiple tissues or organisms**. Except for the development of the initial genome scale reconstructions from various kingdoms of live, only multi-tissue or multi-compartment models are listed. ([1] Edwards and Palsson (1999); [2] Förster et al. (2003); [3] Sheikh et al. (2005); [4] Duarte et al. (2007); [5] Ma et al. (2007); [6] Stolyar et al. (2007); [7] Bordbar et al. (2010); [8] Lewis et al. (2010); [9] Bordbar et al. (2011); [10] Zomorrodi and Maranas (2012); [11] Heinken et al. (2013); [12] Grafahrend-Belau et al. (2013); [13] Cheung et al. (2014); [14] Kumar et al. (2014); [15] Gomes De Oliveira Dal'molin et al. (2015))

While originally flux balance analysis was used on small models, partially due to a lack of knowledge about metabolic pathways and also due to a lack of computational resources, ever more complex models have been constructed over the past years. These models can include multiple tissues within an organism, or even multiple distinct organisms which interact metabolically. We will discuss the creation of these models in the following section and provide an overview of the development of these models in Figure 2.

### 3.1. Constraint based models spanning multiple organisms

While models for isolated microbes are now quite advanced, investigations of interactions between organisms are rare. However, there are multiple examples of interactions, either as symbiotic or mutualistic relations (e.g., plant-mycorrhiza interactions) or as parasites (like human-pathogens). To address these types of interactions and investigate their metabolic effects it is necessary to combine multiple organism networks. The first attempt to perform such a combination of metabolic networks of different species was published by Stolyar et al. (2007). They created a combination of metabolic networks consisting of two symbiotic microbial species and investigated the metabolic exchanges occurring between them. This initial idea has been extended into frameworks for optimization of microbial communities (Zomorrodi and Maranas, 2012; Zomorrodi et al., 2014). A more detailed review was published by Mahadevan and Henson (2012).

The concept of combining different organisms in larger models has also been used in simulating host-pathogen interactions. Bordbar et al. (2010) developed a model of the infection of alveolar macrophages by *Myobacterium tubercolosis* and successfully simulated maximal ATP and nitric oxide (NO) production rates. For the model described by Bordbar et al. (2010), one of the most important constraints was the level of oxygen available to the pathogen, as it is (even in simulations) unable to grow without a minimal amount of oxygen. Furthermore, the original biomass function was adjusted to the conditions in the infected macrophage based on expression data. This was achieved in a step-wise manner. First, random sampling was applied to determine the solution space of each individual metabolic component. Second, linear regression was used by iteratively adding and removing metabolic components to and from the biomass function. This process was performed until a new biomass function could better fit the gene expression data. This led to an altered biomass definition with different metabolic components which could reflect the infectious state of the pathogen. The resulting composition is closer to that observed in the infectious state instead of the phase of maximal growth *in vitro*. The combined model with tailored objective functions was interrogated with respect to changes in ATP, NO, and NADH production fluxes in the macrophage, and in the biomass function flux for both host and pathogen. In addition, *in silico* gene essentiality studies were performed on the combined model and compared to experimental data, showing a better agreement than the same studies on the disconnected pathogen model. Finally, flux changes in both the macrophage and the pathogen were investigated by mapping gene expression data using the GIMME algorithm (Becker and Palsson, 2008).

Recently, Heinken et al. (2013) focused on a model of the interactions of the microbium *B. thetaiotaomicron* and the gut cells of a mouse model and could simulate their growth on five different dietary regimes. The coupling between these models was achieved by creating a new compartment (the intestinal lumen) where the metabolites could be exchanged between the models. This multi-scale model was able to capture the symbiotic growth between mouse and microbe and identify the metabolic crosstalk between the two organisms.

While there have been examples of organism linkage, the main issue present in these attempts is the determination and selection of the links between organisms. Selecting alternative linking compounds could lead to vastly different results and while there are active transporters identified for some compounds, many transport systems allow the exchange of multiple compounds, which makes it difficult to pin down the right selection.

### 3.2. Reconstruction and analysis of multi-tissue models of higher organisms

Coupling models of different tissues within the same organism, or of specific tissues with pathogens is conceptually similar to coupling multiple organisms. Both situations commonly define interactions by allowing the different models to secrete and consume metabolites provided by the external medium or the other model. The main challenges are again to find which compounds are exchanged and determine the extent to which this exchange occurs. If the aim is to model microbial communities, there is commonly the assumption that the aim of all community organisms is to grow. Thus, any interaction between organisms that allows a higher growth rate for both organisms is commonly beneficial for both organisms. In a multicellular organism, with multiple distinct tissues, the aim of each tissue is commonly distinct from growth (with the prominent exception of cancer). Thus, it is important to determine the objectives or required activities of each tissue in a multi-tissue model. Those objectives can include e.g., ammonia detoxification in the liver or energy production in the brain. However, it is also possible to define certain functionalities that have to be provided by each tissue and assume that the general objective is to perform these tasks most efficiently (e.g., with a minimum amount of wasted energy, or a minimal amount of enzymes required). Thus, there are multiple challenges which have to be addressed when trying to model multicellular organisms using multi-tissue models.

One of the earliest multi-tissue models was a two-tissue model of *Arabidopsis thaliana* by de Oliveira Dal'Molin et al. (2010), describing the interactions between mesophyll and bundle-sheath cells. Other models are often considering the different phases of day and night by creating a dual representation of the model (Cheung et al., 2014). As these models are the result of coupling two different models (one for the day and one for the night), they are conceptually similar to multi-scale models. In these models, the interactions between the day and the night model are implemented as reactions which represent the storage of compounds during either the day or the night. The concept was extended to a true multi-tissue model by Gomes De Oliveira Dal'molin et al. (2015) who created this dual representation for roots, stem and leaves. By investigating the exchanges the authors were able to determine the storage compounds transferred between day and night (Cheung et al., 2014) and elucidate the effect of translocation costs between tissues on the localization of biosynthetic activities (Gomes De Oliveira Dal'molin et al., 2015).

The recent advances in modeling multi-tissue models have also been applied to human tissues, starting with a multi-tissue brain model by Lewis et al. (2010). They created three models for different neuron types in the human brain: glutaminergic, γ-aminobutyrate (GABA)ergic, and cholinergic. The models contained the following submodels: a neuron with a neuronal mitochondria, an astrocyte with an astrocytic mitochondria, an endothelium/blood compartment and a interstitial space. The brain model used was based on Recon1 (Duarte et al., 2007), using only reactions indicated to be localized in the brain by the Human Protein Reference Database (Mishra et al., 2006) or by HINV (Yamasaki et al., 2008) and additional reaction evidence based on literature research. The reconstructed models could then be used to investigate downregulated pathways in Alzheimer patients and to obtain a mechanistic overview of the effect of this downregulation. The authors further identified potential routes of acetyl-choline precursor synthesis in the mitochondria. The identification was performed by iteratively removing reactions from Recon1 (Duarte et al., 2007) while retaining acetyl-CoA transport from mitochondria to the cytosol. They could determine three main groups of potential acetyl-CoA biosynthesis using singular value decomposition on a set of 21,000 unique minimal reaction sets obtained by this process. The resulting set included the major two pathways in the generated cholinergic model and allowed the qualitative reproduction of multiple regulatory effects on this specific neuron type.

Subsequently, Bordbar et al. (2011) established a multi-tissue model of adipocyte, hepatocyte and myocyte. In the model by Bordbar et al. (2011), each cell model was reconstructed from Recon1 (Duarte et al., 2007) using the SimPheny toolbox to generate initial draft models followed by manual curation. The integration of these cell models was performed in two steps. The first step was renaming all reactions and metabolites according to the compartments they were localized in. In addition, a new blood compartment representing three different fluids, the interstitial fluid, the urine and the blood, was introduced. All cellular exchange reactions were linked to this novel compartment. The second step of integration consisted of adding a bicarbonate buffer reaction in order to achieve proton balancing in the blood compartment. Biomass functions for each cell type were scaled to the average tissue mass and metabolite degradation in the blood compartment was assumed to be insignificant (Bordbar et al., 2011). The analysis of this multi-scale model was performed using FBA and FVA. When there was more than one objective function, the FBA optimization was performed using a pareto optimality approach. The authors were able to analyze specific interactions (like the alanine cycle and Cori cycles between liver and muscle), and could show that those interactions are indeed necessary.

A model similar to the model constructed by Bordbar et al. (2011) has been described recently (Kumar et al., 2014). While these two models focus on the same tissues, they differ by their method of reconstruction, integration and analysis. Furthermore, Kumar et al. (2014) aimed at obtaining a model for diabetic mice instead of human. However, due to the lack of a genome scale reconstruction of mice at the time, Recon1 was used as a basis for their model. The tissue-specific reconstruction was based on the model building algorithm (MBA; Jerby et al., 2010). However, instead of reconstructing three distinct tissue models, the overall model was reconstructed simultaneously. To achieve this, Recon1 (Duarte et al., 2007), was triplicated to represent all three tissues, and these “tissues” were connected. Subsequently, a high, medium and low confidence set was defined for each part of the triplicated Recon. This extended model was then reduced to its consistent subnetwork (i.e., to a network in which all reactions were able to carry a flux). The resulting model was finally subjected to MBA using the defined confidence sets for the reactions in each “tissue.” While containing the same tissues as the model by Bordbar et al. (2011), it consisted of about four times more reactions. The authors then showed that they could transfer the model predictions on mouse phenotypes to diabetic humans. Validation was performed by comparing model predicted phenotypes to known mouse phenotypes based on OMIM (McKusick, 2007). Expression data was incorporated by adapting the flux bounds according to the expression of the gene. The analysis of the physiological and diseased model was performed with FVA using multiple steps:

1. Reactions affected by gene regulation had their flux bounds set to -100 and 100, or 0 and 100, depending on reaction reversibility, while unaffected reaction flux bounds were equal to -1000 and 1000, or 0 and 1000, again depending on reaction reversibility;
2. An FVA of the physiological condition was calculated;
3. The bounds for the disease states were adapted by doubling and halving the bounds for upregulated and downregulated reactions respectively;
4. The diabetic condition was analyzed using FVA;
5. The FVA results were used to calculate the difference between the two conditions.

The resulting exchange fluxes were compared to phenotypes in the OMIM database and showed a better agreement than the analysis on an unmodified Recon1. Finally the model was analyzed for upregulated and downregulated metabolic pathways based on a contextualized subnetwork. This analysis was again performed using FVA and comparing the obtained fluxes with a randomized regulation, resulting in the indication of several tissue specific upregulated and downregulated pathways, which were in accordance with experimental observations.

The approaches presented show that it has become feasible to simulate multi-tissue systems and that the neglect of metabolic interactions between tissues can hide the true actions of metabolism. However, they also indicate that it is important to define non-trivial objectives for each tissue, or to analyze the qualitative changes in flux potentials in order to obtain useful predictions. The former makes it necessary to define the functions and their extend, which is non-trivial. The latter approach might be a possibility to hint at potential interactions/exchanges between tissues, as the automatic reconstruction will include only those transporters strictly necessary for the functions of the network. Thus, there is much potential in multi-tissue reconstruction that is likely to become addressed in the near future.

## 4. Multi-scale modeling of higher organisms

Up to this point, we have focused mainly on purely stoichiometric models. These models have the advantage of requiring minimal amounts of parameters needing only information about the structure of the underlying network, while still providing the full metabolic capacity. However, they are commonly restricted to simulate non-dynamic processes or compare the situation in two distinct conditions. We will now show recent advances in using constraint based models as parts of larger, multi-scale, frameworks which simulate other cellular processes using dynamic techniques.

The human liver is often a target of metabolic investigations, as it is one of the metabolically most active tissues performing many different tasks. Thus, it comes as no surprise that the metabolic reconstruction of hepatocytes, HepatoNet (Gille et al., 2010), is one of the most frequently employed metabolic networks when attempts at integrating additional processes in human are performed. One of these attempts aimed at integrating regulatory and signaling processes with metabolic networks (Fisher et al., 2013). They used a petri net approach to model the regulatory and signaling network in a dynamic fashion. The petri net was extended by constraint nodes which were linked to the flux bounds in the metabolic model. In addition, objective nodes in the petri net were included, which were used to set the objective in the CBM, and to react to changes in the respective objective values. The CBM was simulated using FBA. The approach employs iterations of the following steps:

1. Calculate the constraint and the objective nodes;
2. Update the metabolic model according to (1);
3. Optimize the metabolic model using FBA;
4. Update the Petri net objective nodes according to the new objective.

Using the generated framework, they investigated bile acid homeostasis, and could generate time courses in agreement with those determined experimentally. In addition, they analyzed genotype-phenotype relationships, from which they could identify several genes which are likely critical in keeping the bile acid homeostasis. The investigation showed a promising way to integrate the effects of regulatory networks on metabolism in a dynamic way. It is however restricted to parts of metabolic networks, where the controlling regulatory network is well understood.

The pharmaceutical industry routinely uses kinetic whole-body models to determine the distribution and effects of drugs on a whole-body scale. These physiologically based pharmacokinetic models (PBPK) aim to describe the absorption, distribution, metabolization and excretion of compounds. They commonly include kinetics for blood flow, and exchange rates for organs or tissues relevant for the biological question. Additionally, they are based on prior knowledge of anatomy, physiology as well as of compounds properties. Furthermore, they can give rise to an understanding of the macroscale effects of a drug treatment. Thus, combining these large scale models with the cellular models above provides the potential to understand both whole-body behavior and individual cell responses and can provide information on the potential side effects of a treatment. This concept has been applied to human (Krauss et al., 2012) and arabidopsis (Grafahrend-Belau et al., 2013) and we will discuss both studies in more detail. In the system developed by Krauss et al. (2012) a liver CBM and a whole-body physiologically based pharmacokinetics (PBPK) model were coupled. In order to study, how these compounds affect cellular metabolism, the CBM models were integrated, replacing parts of the tissue specific functions. The model chosen to serve as a CBM was Hepatonet, which was simulated by a static dFBA approach. Since this static method uses linear programming, it is not computationally expensive. The models were coupled using two different approaches. Indirect coupling was used to simulate processes that have no direct effect on the PBPK model e.g., administration of a compound that affects metabolic enzyme activity. For these processes information from the PBPK model was used to constrain the CBM model. For processes that have a direct influence on the dynamics of the PBPK model, direct linkage was used. To achieve this coupling, a feedback update loop was integrated in order to update the fluxes for the next time step in the PBPK model according to those resulting from the simulation of the CBM model. This feedback loop consists of four steps:

1. Clearance and production rates are calculated at the whole-body level using the PBPK model;
2. The upper bounds of coupled reactions in the CBM are adjusted to the calculated values;
3. The CBM is simulated and the coupled rates are determined;
4. The directly coupled PBPK rates are set to the rates obtained in 3 to determine the metabolite levels for the next iteration.

Indirect coupling was used to simulate both the effect of allopurinol treatment on hyperuricemia and a paracetamol overdose causing liver toxification. Direct coupling of the models was employed to simulate impaired ammonia detoxification, where the activity of a specific enzyme in the CBM was impaired. The modeling approach can be applied to study different processes, inferring the effect of cellular alterations on the whole-body level. However, it does not allow a direct assessment of the cellular flux distribution, as the objective used in the CBM model is to maximize the coupled fluxes while minimizing the remaining fluxes, which can lead to odd internal flux distributions.

In an approach similar to the one used by Krauss et al. (2012) and Grafahrend-Belau et al. (2013) were simulating a multi-scale model of barley. The multi-tissue plant model was reconstructed from literature-based biochemical reactions and databases. It included leaf, stem, seed, and root models which were coupled by adding a phloem compartment that was used for the exchange of carbon and nitrogen sources between the different plant compartments. The multi-tissue model was coupled to the dynamic whole-plant ProNet-CN model (Mueller et al., 2012). In order to analyze the multi-tissue model, dynamic FBA was used. The optimization of the multi-tissue model was performed in two steps: first the carbon uptake was minimized and the result was used to constrain the second optimization step which consisted of a minimization of all fluxes using the minimal carbon uptake. The coupling approaches were similar to those used by Krauss et al. (2012). The resulting modeling framework was then used to study the seed developmental phase of barley plants.

As could be seen, there are many approaches, to add additional levels of cellular processes to constraint based models, or use constraint based models as parts of complex whole organism simulations. And while recently a whole-cell model of *Mycoplasma genitalium* has been published by Karr et al. (2012), this model requires over 1900 parameters to be fitted or determined. While this has been achieved on a prokaryotic, rather “simple” system, the same would require many more parameters for eukaryotic cells and likely at least an order of magnitude more for multicellular organisms, exhibiting multiple types of tissues. Thus, approaches with simplified processes, like CBM models, will serve as a good basis for quite some time to come, but as we showed, it is always possible to combine them with other types of models, to improve our capabilities in simulating the effects of metabolic interventions on larger systems.

## 5. Conclusion

Even with our constantly growing understanding of the kinetic properties of enzymatic processes, the multitude of different conditions and slight variations makes it unlikely that a multi-cellular kinetic model comprising all known metabolic pathways will be established in the near future. Thus, we need modeling techniques that can cope with the size of a complex multi-tissue network. We have presented several examples of purely constraint based modeling approaches that aim at providing these techniques. These methods however, lack the capability to include effects like distribution limits, or the dynamic response to the changes in metabolite levels generated by the CBM models. In the recent years, methods to combine CBM models with other types of models have been proposed to address these issues. These techniques could allow us to achieve a better understanding of the interactions between dynamic processes on the whole-body scale, and cellular metabolic processes. They provide ways to integrate additional levels of control influencing the behavior of the metabolic network, and could provide means to decipher the intricate interactions of the different regulatory processes in a higher organism. Thus, further development and improvement of these techniques is likely to be an important step in our voyage to a complete understanding of metabolism.

## Author contributions

TP and TS designed the study. PM, TP, and TS wrote, edited, and refined the manuscript.

## Funding

PM, TS, and TP are supported by the University of Luxembourg.

### Conflict of interest statement

The authors declare that the research was conducted in the absence of any commercial or financial relationships that could be construed as a potential conflict of interest.
